# Broken by the Cut: A Journey into the Role of Topoisomerase II in DNA Fragility

**DOI:** 10.3390/genes10100791

**Published:** 2019-10-12

**Authors:** Naomi D. Atkin, Heather M. Raimer, Yuh-Hwa Wang

**Affiliations:** Department of Biochemistry and Molecular Genetics, School of Medicine, University of Virginia, Charlottesville, VA 22908, USA; nda2dj@virginia.edu (N.D.A.);

**Keywords:** DNA topoisomerases, DNA fragility, chromatin organization, TOP2 cleavage complex, topoisomerases and disease, DNA repair

## Abstract

DNA topoisomerase II (TOP2) plays a critical role in many processes such as replication and transcription, where it resolves DNA structures and relieves torsional stress. Recent evidence demonstrated the association of TOP2 with topologically associated domains (TAD) boundaries and CCCTC-binding factor (CTCF) binding sites. At these sites, TOP2 promotes interactions between enhancers and gene promoters, and relieves torsional stress that accumulates at these physical barriers. Interestingly, in executing its enzymatic function, TOP2 contributes to DNA fragility through re-ligation failure, which results in persistent DNA breaks when unrepaired or illegitimately repaired. Here, we discuss the biological processes for which TOP2 is required and the steps at which it can introduce DNA breaks. We describe the repair processes that follow removal of TOP2 adducts and the resultant broken DNA ends, and present how these processes can contribute to disease-associated mutations. Furthermore, we examine the involvement of TOP2-induced breaks in the formation of oncogenic translocations of leukemia and papillary thyroid cancer, as well as the role of TOP2 and proteins which repair TOP2 adducts in other diseases. The participation of TOP2 in generating persistent DNA breaks and leading to diseases such as cancer, could have an impact on disease treatment and prevention.

## 1. Introduction

Topoisomerases relieve supercoiling in a sequence-independent manner by transiently cutting the DNA, passing the DNA strand through the break, and then re-ligating the strand(s) [1,2]. The two major groups/types of topoisomerases are type I and type II, with type I enzymes generating single-stranded breaks and type II enzymes generating double-stranded DNA breaks (DSBs) [1,2]. Two isoforms of type II topoisomerases, topoisomerase IIα (TOP2A) and topoisomerase IIβ (TOP2B), are active across much of the genome and throughout the cell cycle [3].

Recently, Hoa et al. demonstrated that TOP2 frequently fails to complete re-ligation, resulting in a persistent TOP2 cleavage complex (TOP2cc) that needs to be processed to prevent interference with replication and transcription machineries [4]. Along with the inherent errors of TOP2, there are also a variety of endogenous and exogenous factors that can impair TOP2 function. Catalytic inhibitors of TOP2, such as merbarone [5,6], prevent the initial TOP2 cleavage of DNA, while TOP2 poisons prevent the re-ligation by stabilizing the TOP2cc state. Common TOP2 poisons include chemotherapeutic drugs (etoposide and doxorubicin) [7,8], environmental chemicals (benzene) [9,10,11], dietary factors (bioflavonoids) [12], and endogenous stressors (base mismatches and apurinic sites) [13,14,15,16]. By stabilizing TOP2ccs, TOP2 poisons also promote the persistence of DSBs generated in the process [17]. DSBs induced by exposure to exogenous TOP2 poisons are suggested to occur at similar locations as endogenous breaks; therefore, the exposure increases the likelihood that the DSB is converted into a persistent break [18,19]. Thus, the consequences of TOP2-induced DSBs are dependent on where the breaks are generated, both endogenously and in response to external stressors, and how the TOP2ccs are removed and repaired.

## 2. TOP2 Cleavage in Multiple Biological Processes:

### 2.1. TOP2-Induced Breaks Contribute to Replication-Associated DNA Fragility

**Figure 1:**
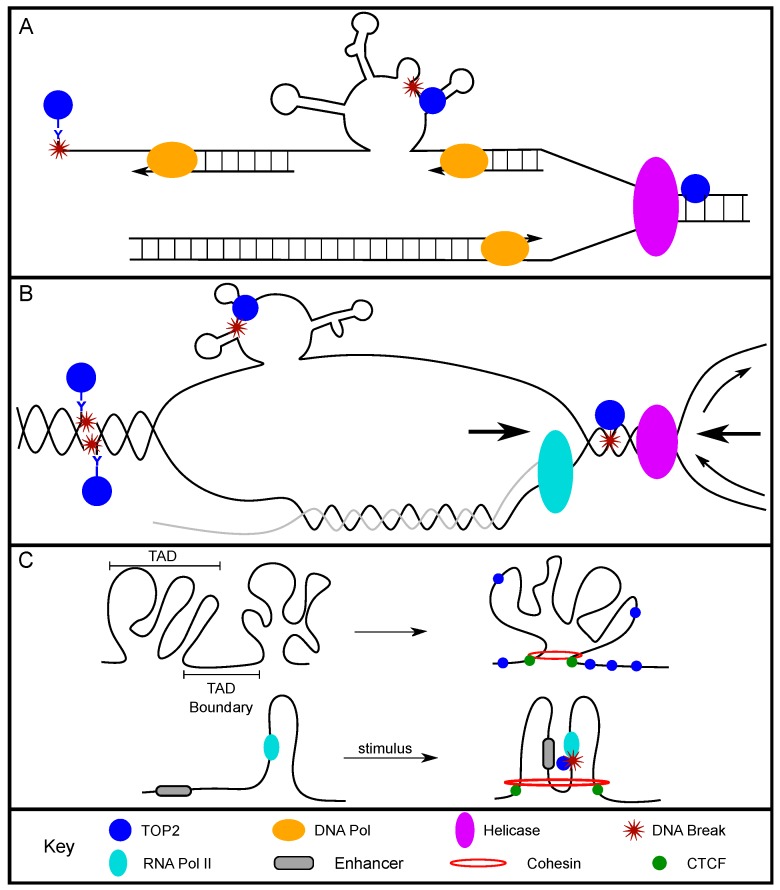
DNA topoisomerase II (TOP2) cleavage is required for multiple biological processes. (**A**) During DNA replication, TOP2 relieves positive supercoiling ahead of the replication fork. Single-stranded DNA on the lagging strand template can form alternative DNA secondary structures that can become targets of TOP2. Furthermore, if a TOP2 cleavage complex (TOP2cc) already exists in replicating DNA, collisions between DNA polymerase and the adduct generate DNA breaks. (**B**) During transcription, TOP2 resolves negative supercoiling behind RNA polymerase II (Pol II), which if re-ligation fails could create TOP2ccs. Also, the non-template strand can form alternative DNA secondary structures, which become another target for possible TOP2 cleavage. There is also the possibility of transcription and replication collisions which can cause breaks potentially mediated by TOP2 acting on the high degree of supercoiling at these collisions. (**C**) TOP2 is also involved in the maintenance of chromatin organization, where it relieves supercoiling that occurs around CCCTC-binding factor (CTCF)- and cohesin-defined topologically associated domain (TAD) boundaries. Additionally, when stimulation activates transcription by promoting the interaction between an enhancer and a regulated gene, TOP2 is associated with DNA breaks at transcription start sites (TSSs) of the activated genes.

TOP2 was found to relieve supercoiling at origins of replication in order to preserve the underwound state of the DNA, but it does not promote replication initiation itself [20,21,22,23]. TOP2 also plays an active role in replication elongation [24]. The positive supercoiling that accumulates in front of the replication fork/DNA polymerase is a preferential substrate for TOP2A [25]. TOP2 can also resolve DNA precatenane structures behind the fork, relieve the extremely high positive supercoiling between two converging replication forks, and promote replication fork fusion at termination regions [26,27,28]. These studies show strong support for the role of TOP2 in resolving structures and torsional stress at and between replication forks, and provide possibilities for the presence of TOP2-induced breaks at these sites (Figure 1A).

Studies demonstrated that the cytotoxicity of TOP2 poisons, such as treatments with etoposide or amsacrine, was reduced when cells were co-treated with aphidicolin, an inhibitor of the replicative DNA polymerases α, δ, and ε [29,30]. This suggests that the TOP2 poison-related cytotoxicity was due to replication forks colliding with stable TOP2ccs, which generated persistent DSBs (Figure 1A). Treatment with low concentrations of etoposide produced more discrete replication protein A (RPA) foci mainly in S-phase nuclei of U2OS cells compared to untreated cells [31]. As RPA foci can be formed at helicase-unwound single-stranded DNA (ssDNA) sites of DNA replication and 3′ ssDNA resected from DNA damage sites, the increased foci supported the idea that the DNA was resected [32,33]. Furthermore, when etoposide-treated cells were exposed to aphidicolin, there was a marked reduction in RPA foci-positive cells, indicating that replication elongation was required for the accumulation of etoposide-induced RPA foci, possibly due to replication collision with TOP2ccs [31]. Our group demonstrated the involvement of TOP2 in replication-associated DNA fragility at fragile sites. We found that, when non-malignant human thyroid cells were treated with aphidicolin (a classic fragile site-inducing chemical), *RET* (a non-transcribed gene in thyroid cells located within FRA10G) and *FHIT* (within FRA3B) had more aphidicolin-induced DSBs compared to non-fragile control regions. More importantly, when these cells were treated with both aphidicolin and a TOP2 catalytic inhibitor, merbarone, the replication-associated breaks were significantly reduced [34]. This directly demonstrated that TOP2 cleavage mediated the replication-associated DNA fragility at these regions. Overall, these studies support the role of TOP2, and specifically the formation of stable TOP2ccs, in replication-dependent DNA fragility. This could provide an opportunity for TOP2 to introduce persistent DSBs during replication, whether through failure to re-ligate the DNA strands or through replication TOP2cc collision and subsequent fork collapse. Therefore, TOP2 plays a direct role in mediating replication-associated DNA fragility.

### 2.2. TOP2-Induced DNA Breaks and Transcription Activation

During active transcription, DNA strand separation generates positive supercoiling ahead of the RNA polymerase and negative supercoiling behind the polymerase [35] (Figure 1B). Importantly, the status of DNA supercoiling can greatly impact transcription. A study using yeast showed that positively supercoiled DNA inhibited transcription, observed as decreased messenger RNA (mRNA) production [36]. Conversely, negatively supercoiled DNA can promote transcription by allowing for promoter melting and recruitment of initiation-associated proteins, as well as recruitment of RNA polymerase II (Pol II) [37,38,39,40]. TOP2, as a modulator of the supercoiled state, can play a direct role in regulating these dynamic transcription states.

Many studies have examined the role of TOP2 in transcription in human cells. Genome-wide chromatin immunoprecipitation and sequencing (ChIP-seq) analysis by Manville et al. found that 50% of TOP2B peaks were located in genes or within 5 kb of transcription start sites (TSS) [41]. Importantly, other studies showed that TOP2-induced DNA breaks facilitate transcription activation, especially among early developmental genes and estrogen- or androgen-response genes. The recruitment of TOP2 to the promoter and TSSs of these genes was demonstrated to couple with the accumulation of the DSB-associated protein γH2AX at these sites following hormonal or neuronal stimulation [42,43,44,45]. Moreover, it was shown that TOP2 cleavage was directly required for the stimulus-induced DNA damage observed in these regions, as the addition of catalytic inhibitors, merbarone or ICRF-193, abrogated formation of γH2AX foci or DNA break products [42,44,46,47]. Specifically, Trotter et al. observed DNA breaks on both strands of the promoter following glucocorticoid stimulation, further supporting the role of TOP2 cleavage at gene promoters and TSSs [47]. In addition, treatment with TOP2 poisons caused increased transcript levels of these genes, which indicates that TOP2-induced DSBs promote efficient signal-induced transcription [43,48]. Recent genome-wide direct break mapping/sequencing studies further established that the TSS and promoter regions of highly expressed genes are enriched for TOP2-induced DNA breaks [18,19,49], supporting the notion that TOP2-induced DNA breaks facilitate transcription activation. However, transcription- and TOP2-induced DSBs can result in pathological abnormalities such as chromosomal translocations, as evidenced by *TMPRSS2–ERG* fusions that occur following androgen-mediated transcription of these genes in prostate cells [45]. Translocations were also shown to occur following transcription-associated DSBs in a primary mouse neural stem/progenitor cell model, in which active genes with translocation junctions within 2 kb of TSSs were found to have significantly higher expression than those with junctions in the gene body [50]. These studies highlighted the critical impact of TOP2-induced DSBs in transcription and the delicate balance between activation of transcription and the generation of chromosomal abnormalities.

Furthermore, in a subset of large genes (>800 kb), replication and transcription must occur simultaneously for proper gene replication. Such instances increase the likelihood of replication and transcription collision, resulting in DNA breaks and genomic instability (Figure 1B) [51]. The transcription of long genes that occurs in S phase especially requires the activity of TOP2 [52,53,54]. Notably, two long genes, *FHIT* and *WWOX,* often exhibit deletions within collision-associated intronic regions in cancer cells [55,56,57,58,59]. Therefore, the deletions that are observed in cancer cells could be the result of faulty repair processes following TOP2-induced DSBs during transcription–replication collisions. Ultimately, TOP2 is required to facilitate efficient transcription and replication elongation, and consequently introduces DSBs that, if illegitimately repaired, can become translocations or deletions.

### 2.3. TOP2 and Chromatin Organization

The human genome is organized and compacted into large structures called topologically associated domains (TADs) [60,61]. Boundaries of TADs have a higher density of housekeeping genes and the architectural protein CCCTC-binding factor (CTCF), and the boundary regions contribute to the regulation of gene expression and overall chromatin organization [60,61]. In a study done in human retinal pigment epithelial cells, negatively supercoiled domains were enriched for DNase-I hypersensitivity, TSSs, RNA polymerase, and were highly transcribed and within 20 kb of CTCF-binding sites [62].

TOP2 acts at TAD boundaries/CTCF-binding regions to alleviate torsional stress and/or to allow for regulatory elements to be brought into proximity with one another (Figure 1C). Sano et al. found that the expression of a subset of neuronal genes was dependent upon TOP2B activity by examining the genome-wide location of functional TOP2B and gene expression data. This study suggests that the surrounding AT-rich region created a chromatin structure that required TOP2B to relieve repression of gene expression [63]. Madabhushi et al. further supported this when they found that neuronal activity induced TOP2B-dependent DSBs [43]. The identified DSBs were localized to the promoters of early-response genes, and promoted gene expression in neurons. Importantly this study showed that the TOP2B binding sites found in both basal expression and post-neuronal stimulation, were enriched with CTCF motifs. The direct interaction between CTCF and TOP2B was further characterized and found to be enhanced following neuronal stimulation [43]. Supporting these findings, Uuskula-Reimand et al. showed colocalization of CTCF, TOP2B, and RAD21 at TAD boundaries in mice [64]. Highly conserved CTCF-binding sites, both across cell types and across species, colocalized with TOP2B and RAD21 binding sites. The order of protein binding along the DNA was determined, with TOP2B as the most 5′ and RAD21 as the most 3′ from the center of the CTCF motif. Furthermore, the DNA at the CTCF motifs was negatively supercoiled, and RNA Pol II or TOP2 inhibition caused the DNA to exist in either an overwound or neutral supercoiled state [64]. Altogether, these studies establish that TOP2 crucially functions at TAD boundaries/CTCF-binding sites and is capable of impacting supercoiling and gene expression.

Using genome-wide break mapping/sequencing methods such as END-seq and BLISS, TOP2-induced breaks were directly measured and indeed were concentrated at CTCF-binding sites [18,19,65]. These studies found that TOP2B-induced breaks were dependent upon transcription, and highly expressed genes were more sensitive to this class of breaks [18,19]. Canela et al. also found that TOP2-induced DSB peaks had an increase of enhancer-promoter loops. Furthermore, 63% of loop anchors contained etoposide-induced DSB peaks on both sides of the CTCF-bound border as a result of topological stress [65]. CTCF-anchored loops with TOP2B-dependent DSBs had significantly higher loop interactions compared to loops that did not possess TOP2B-dependent DSBs [65]. Naughton et al. showed that the enzymatic activity of topoisomerases at TAD boundaries and loops is necessary to bring two segments of DNA into closer proximity with one another [62]. These studies suggest that topological stress at CTCF-bound regions of the genome prompts TOP2 to bind and introduce breaks. This can then promote gene expression by facilitating closer interactions between enhancers and promoters.

While frequent interactions within TADs promote transcription, these interactions can be detrimental if the two segments are incorrectly joined together and result in an oncogenic translocation. Previous studies showed that translocation-associated genes, specifically *MLL* and its partner genes *AF9* and *AF4*, undergo transcription within the same transcription factories, and are, therefore, in close proximity [66]. Furthermore, in activated B cells from mice, loop anchors (regions bound by CTCF, RAD21, and TOP2B) had an enrichment of TOP2-induced DSB peaks, as compared to random regions of the genome, and these DSB-enriched loop anchors also included translocation breakpoint cluster regions like *Mll, Af9, Af4,* and *Enl* [65]. Using a four-color break-apart fluorescence in situ hybridization (FISH) assay, Gothe et al. demonstrated that translocations between *MLL* and its partner genes (*AF4, AF6, AF9,* and *ENL*) significantly increased upon etoposide treatment of lymphoblastoid cells [18]. Using a murine leukemia model, Canela et al. also found that translocations were formed and preferentially presented in TOP2-induced DSB peaks. This enrichment was dependent upon transcription, and translocations occurred more often in highly transcribed genes [19]. Therefore, TOP2 has a role in both transcription and chromatin organization, and both processes, if not properly regulated, can further contribute to opportunities for oncogenic translocations involving TOP2-induced DSBs.

## 3. Alternative DNA Secondary Structures and Unusual Bases Promote TOP2-Induced DSBs

**Figure 2:**
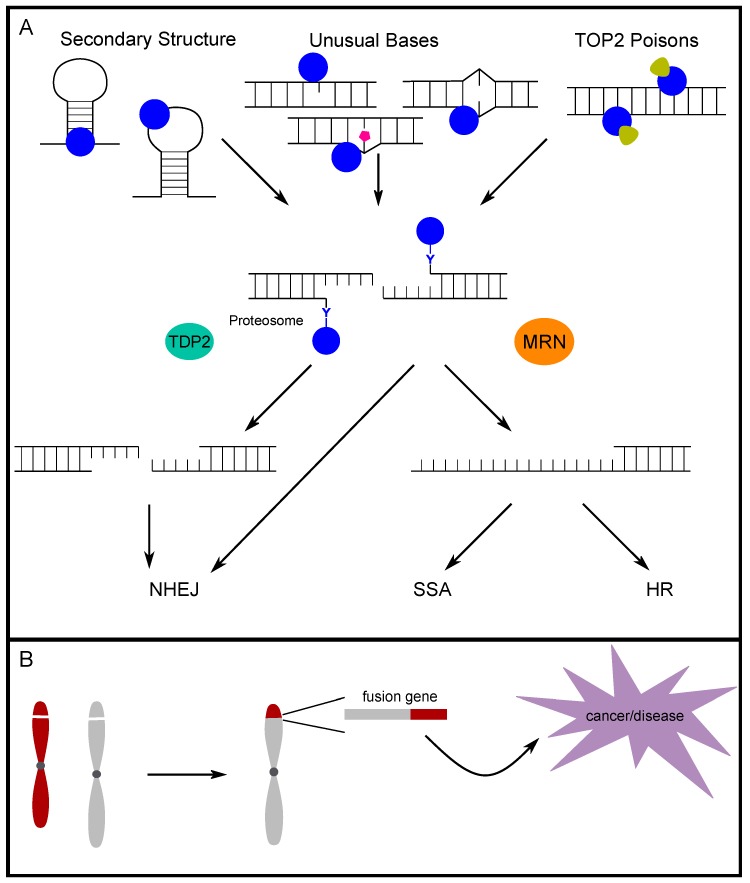
Targets and stabilizers of TOP2 cleavage, repair pathways, and consequences of improper repair. (**A**) Alternative DNA secondary structures, such as hairpins, can be targets of TOP2 (blue) cleavage both at single-stranded loops and bases of stems. Mismatched bases, abasic sites and sites with bulky adducted bases, also serve as TOP2 targets and act as endogenous TOP2 poisons stabilizing TOP2ccs. Furthermore, many exogenous TOP2 poisons are capable of stabilizing cleavage complexes. Persistent TOP2ccs are removed either through a TDP2-dependent pathway or through an MRE11 (MRE11/RAD50/NBS1; MRN) pathway. TDP2 works following proteosomal degradation of TOP2 and removes the final tyrosine-bound nucleotide. The non-homologous end-joining (NHEJ) pathway repairs the TDP2-modified ends. When MRE11 (MRN) is employed in repair of TOP2ccs, end resection occurs and triggers repair pathways, non-homologous end-joining (NHEJ), single-strand annealing (SSA), or homologous recombination (HR), dependent on what proteins are further recruited and the extent of end resection. (**B**) Consequences of unrepaired or illegitimately repaired DSBs on human chromosomes can lead to gene fusion and rearrangement events which underlie cancers and other diseases.

TOP2 responds to torsional stress generated in the maintenance of the genome; however, supercoiling is not required and TOP2 does not have a stringent recognition sequence [67,68]. Interestingly, there is evidence that alternative DNA secondary structures are targeted by topoisomerase for enzymatic cleavage (Figure 2A) [69,70,71,72]. Specifically, Froelich-Ammon et al. demonstrated that TOP2 can recognize and cleave stem-loop structures one nucleotide from the 3′ base of the stem structure [69]. Additionally, TOP2 was shown to cleave the single-stranded loop of stem-loop structures formed in alpha-satellite sequences. This activity is seen in the alpha-satellite contained in centromeres across multiple species, which further points to a non-B DNA secondary structure as the common site for TOP2 cleavage [70,71]. Furthermore, West and Austin demonstrated that TOP2B not only binds and cleaves four-way junction DNA, but also has on average a four-fold higher affinity for four-way junction DNA compared to linear DNA [72]. Our group previously mapped breaks occurring in intron 11 of *RET* upon induction of replication stress via treatment with aphidicolin. Not only did the breaks map closely to topoisomerase cleavage sites, but they also mapped to bases of stem and loop regions of the most stable predicted secondary structures for the region, which exactly matches the structural features of TOP2 recognition [34]. Additionally, when we mapped TOP2 cut sites in vitro, the same sites were identified. Furthermore, we validated the predicted structure-forming ability by demonstrating the formation of alternative DNA secondary structure by the *RET* intron 11 sequence in vitro, and expanded this computational analysis across multiple fragile sites in chromosome 10 and the genome [73,74]. These analyses show that fragile sites are prone to forming clustered regions of highly stable DNA secondary structures, potential targets of TOP2 cleavage, which implicates TOP2 in fragile site instability [75]. Analyzing translocation breakpoints in the *MLL* gene, Le et al. identified a break cluster region with a potential to form a stem-loop structure. They proposed that TOP2 could bind the region and facilitate the formation of secondary structure, and that DNA breaks can be generated by TOP2 and/or other structure-specific nucleases [76]. We [77] subsequently showed in human hematopoietic stem/progenitor cells that etoposide- and doxorubicin-induced DNA breaks were indeed enriched at the translocation break cluster region found in patients, and this region is energetically favorable to form multiple stem-loop structures and includes the region identified by Le et al. These studies suggest that whether through the direct cleavage of secondary structures by TOP2, the facilitation of secondary structure formation by TOP2 and eventual targeting by other structure-specific nucleases, or a combination thereof, TOP2 contributes to genomic instability via secondary structures.

Abasic and mismatched sites in double-stranded DNA are suggested to be endogenous “poisons” to TOP2 (Figure 2A) [13,14,15,16]. Bigioni et al. investigated the impact of mismatched bases on TOP2 activity and found that, when a mismatch existed in the 4-bp TOP2-induced overhang, specifically mismatches at the +1 and +2 positions, TOP2 cleavage was increased to a greater extent than with etoposide treatment [15]. To further understand endogenous TOP2 poisons, Kingma et al. investigated the impact of abasic sites in similar positions as in the previous study. All forms of endogenous damage studied were found to promote the activity of TOP2, but apurinic sites had a particularly potent ability to increase TOP2 cleavage. Apurinic sites were able to induce TOP2 cleavage to a similar, or even greater, extent than TOP2 poisons such as etoposide [13]. To understand how abasic sites and mismatches function as “endogenous TOP2 poisons”, Cline et al. demonstrated that an oligomer with similar apurinic sites resulted in a distortion in the helical backbone, bringing the scission sites on each strand closer together, which could reduce the activation energy for TOP2 cleavage and promote more breaks [14]. Sabourin and Osheroff investigated other naturally occurring lesions such as oxidative and alkylating damage, and found that 1,*N*^6^-ethenoadenine lesions, which cannot base-pair and create a distortion in the DNA backbone, also promoted TOP2 cleavage [16]. In an experiment to determine long-range targeting, 1,*N*^6^-ethenoadenine lesions caused a similar degree of cleavage promotion as apurinic sites [16].

Altogether, these studies support that helical distortions are a requirement and likely provide an energetically favorable target and, therefore, can be a mechanism for the endogenous generation of TOP2 breaks. Both secondary structure formation and these internal DNA lesions regularly occur in healthy cells [78,79], presenting targets for TOP2 cleavage. The processes of transcription, replication, and maintenance of chromatin loops can provide the single-stranded DNA necessary for secondary structure formation. This introduces an intriguing possibility that alternative DNA secondary structures, or lesions, serve as major contributors for breaks during these genomic processes. Our group demonstrated that alternative DNA secondary structures are enriched at TSSs, and that these structures lead to RNA Pol II promoter-proximal pausing, with a greater degree of pausing correlated with more energetically favorable structure formation [74]. TOP2 travels with RNA Pol II during transcription, and the presence of structures at TSSs provides a target for TOP2 activity. Indeed, Bunch et al. demonstrated TOP2-induced release of RNA Pol II promoter-proximal pausing promoted transcription elongation [44]. Dellino et al. further confirmed that TOP2 is necessary for release of paused RNA Pol II and determined that the degree of pausing and the recruitment of TOP2 to paused sites was a major determinant of DSB formation at the locus [80]. These findings can explain, at least in part, the observations of increased DSBs located at TSSs [81,82]. The presence of alternative DNA secondary structures and DNA lesions promote TOP2 activity at sites across the genome, which can lead to damage at these sites if TOP2 fails to complete re-ligation. However, the ultimate outcome of a failed TOP2cc lesion is dependent on whether the TOP2cc is resolved and what pathways are used to repair the damage.

## 4. The Fate of TOP2-Induced Breaks

### 4.1. TOP2-Adduct Removal and End Processing

Whether TOP2ccs occur endogenously or in response to exogenous stressors, two major pathways, TDP2 (tyrosyl-DNA phosphodiesterase 2) and MRE11 in the MRE11/RAD50/NBS1 (MRN) complex, were shown to process and resolve TOP2cc adducts in human cells (Figure 2A).

The TDP2 pathway removes TOP2ccs in a paired process in which proteasomal degradation of TOP2 leaves behind only the 5′ tyrosyl group that is covalently bound to the end of DNA [83]. This 5′ tyrosyl moiety is then removed by TDP2, which removes only the nucleotide that is bound by the tyrosine. The 5′ tyrosyl DNA phosphodiesterase activity was initially described in 2009 [84]. Subsequently, the impact of TDP2 was determined in mammalian cells [85]. Zeng et al. found that TDP2 had specificity for 5′ tyrosyl DNA ends and further demonstrated that this activity was a major factor in cell survival following TOP2 poison treatment [85]. When TOP2ccs are removed by TDP2, a very limited amount of end processing at the break results in a clean, ligation-compatible end that can then be repaired almost exclusively through the non-homologous end-joining (NHEJ) repair pathway [86,87]. The ability of TDP2 to act and repair TOP2ccs caused by TOP2 poisons suggests that the TDP2 status of patients may be an important factor to consider when considering treatment with TOP2 poisons [85,87,88].

The MRE11 pathway is able to remove the entire moiety of TOP2cc, and this can occur before proteasomal degradation [89]. As a member of the MRN complex, MRE11 has both endo- and exonuclease (3′ to 5′) activities. The activity of MRE11 in the MRN complex leads to the removal of the 5′ TOP2 adduct. The process via which MRE11 relieves a 5′ TOP2 adduct was demonstrated in vitro with two variations: (1) directly cutting approximately 18 nt beyond the adduct on the same strand followed by exonuclease digestion moving toward the adduct, and (2) creating an endonuclease nick on the non-adduct strand followed by exonuclease degradation away from the adduct end and finally, another endonuclease cut at the double strand–single strand boundary, thus releasing the 5′ adduct DNA [90]. The nuclease activity of MRE11 during removal of TOP2ccs (and general 5′ adducts) was shown to be regulated by CtIP (CtBP1 interacting protein)-BRCA1 (breast cancer type susceptibility protein) [91]. CtIP was required to successfully clear TOP2 adducts that accumulated during replication in *Xenopus* egg extracts. Furthermore, the proper phosphorylation for binding with BRCA1 was required for resection of 5′- blocked DNA ends by MRE11 [91]. Additionally, NBS1 (nibrin; in the MRN complex) provides control of the MRE11 nuclease activity by sensing other phosphorylation events on CtIP. This is shown to limit more extensive resection by MRE11 to the S/G2 phases of the cell cycle when repair can be directed to homologous recombination (HR) [90,92]. Although MRE11 canonically leads to the HR repair pathway, MRE11 also processes TOP2ccs during G1 and can direct repair to NHEJ [93]. MRE11-mediated removal of TOP2ccs in G1 was also found to be CtIP-dependent when etoposide stabilized TOP2cc formation [93]. Thus, MRE11 is able to assist in the processing of TOP2ccs throughout the cell cycle and is able to shunt repair to either NHEJ or HR dependent upon interactions with NBS1, CtIP, and BRCA1 [94]. The ultimate repair of TOP2ccs and which pathway is used to complete repair, can have important implications for genome stability. Therefore, a better understanding of TOP2cc processing is critical to reveal the impacts of TOP2 on genome integrity.

### 4.2. Determining End Structures and Repair Intermediates

The different pathways that remove TOP2cc adducts result in different repair intermediates that could be captured as TOP2-induced breaks. Recently, genome-wide DSB mapping/sequencing methods that are capable of single-nucleotide resolution were developed [81,82,95,96,97] (further reviewed in References [49,97]). To take advantage of the single-nucleotide resolution break mapping data, we developed an analysis tool, coverage-normalized cross correlation (CNCC). CNCC uses the single nucleotide encoding a DNA break and strandness of each mapped break nucleotide to assess the overall genome-wide break-type profile in a given cell line or after a given treatment [98]. The CNCC analysis, on a genome-wide scale, can distinguish the three major DNA end structures: blunt-ended, 3′ overhangs, and 5′ overhangs without a priori knowledge of break sequences or genomic position. Following treatment with the TOP2 poison etoposide, CNCC revealed a global pattern of 3′ overhangs, indicating 5′ to 3′ resection activity likely resulting from repair of TOP2ccs. Additionally, this resection signature increased and progressed further upon treatments with higher concentrations of etoposide, suggesting a robust response to damage induced by repair of TOP2ccs. While the extent of resection was thoroughly determined in yeast, and explored at specifically induced break sites in mammalian cells, our CNCC analysis is the first to reveal the extent of TOP2-induced resection in cells at a genome-wide scale [33,99]. Distinguishing the repair intermediates following etoposide treatment allows for greater insight into what repair mechanisms are employed. While the peak at short-range resection could be repaired by NHEJ, the longer-range resection that becomes more prevalent in higher-concentration treatments indicates that a push toward HR occurs, possibly due to a higher damage burden being present during S/G2.

### 4.3. Repair Pathway Choice for TOP2-Induced Breaks

The decision for what pathway is used to repair DSBs is influenced by a combination of DNA end structure, proteins recruited to the DNA ends, and cell-cycle stages. When TOP2ccs are removed by TDP2, only the nucleotide that is bound to the tyrosine is removed, leaving ligation-compatible ends that can undergo NHEJ. The Ku complex recognizes the ends and p53 further promotes NHEJ by blocking HR-related proteins. The ligation is then carried out by the XRCC4/LigIV complex for simple, compatible ends [100]. TOP2cc removal and repair is more complex when MRE11 is involved. In this case, TOP2-adducted ends receive a very limited processing to nick away a fragment with TOP2 still attached. Subsequently, the recruited proteins and cell cycle ultimately determine which repair pathway is followed by controlling whether long-range resection occurs. When short-range resection occurs, but longer-range resection is not employed, both the alternative NHEJ (A-NHEJ) and single-strand annealing (SSA) pathways can repair TOP2-induced breaks [101]. While, in G1, the repair is more likely to go through NHEJ, A-NHEJ, or SSA, in S/G2, HR becomes available, since proteins for long-range resection have the appropriate post-translational modifications (Figure 2A) [33,101,102,103]. Major players of control through the cell cycle are cyclin-dependent kinases and ataxia-telangiectasia mutated (ATM), which control post-translational modifications of many of the key repair proteins leading to a switch between states throughout the cell cycle [101,104,105]. Therefore, genomic integrity is dependent both on the appropriate function of TOP2 and proteins involved in the repair of TOP2 lesions. When repair is improperly completed, deletions, insertions, or translocations could occur and, if located in crucial genomic regions, can lead to a variety of human diseases (Figure 2B) [79].

## 5. The Role of TOP2-Induced Breaks in Disease

### 5.1. Therapy-Related Acute Myeloid Leukemia

TOP2 poisons are commonly utilized in standard chemotherapy regimens. The active role of TOP2 in various biological processes enables TOP2 poisons to be incredibly effective in cancer treatment, as the resulting fork collapses/collisions and unresolved DSBs lead to apoptosis of the cancer cells. However, a common consequence of this mode of action is DNA damage to normal cells, as demonstrated by increased sensitivity of hematopoietic stem and progenitor cells (HSPCs) to DNA damaging agents when compared to more committed progenitor cells [106,107]. Heightened sensitivity and subsequent DSBs can then cause mutations that promote secondary malignancies, such as therapy-related acute myeloid leukemia (t-AML). Notably, breast cancer and non-Hodgkin’s lymphoma (NHL) patients treated with the TOP2 poisons, daunorubicin, etoposide, and doxorubicin, are those with the highest risk of being diagnosed with t-AML [108,109,110]. The incidence of t-AML was reported up to 6.3%, with a median occurrence of 3–5 years following standard dose chemotherapy [111,112,113,114], and the incidence of t-AML after high-dose chemotherapy increased to 8.6–13.1% [115,116]. Therefore, it is critical to understand the processes that contribute to the formation of t-AML driver mutations.

Interestingly, the mutation most often associated with t-AML is a translocation involving chromosome 11, specifically the *KMT2A* or *MLL* gene [114,117,118,119]. In 110 t-AML cases, 66% of patients had *MLL* fused to either *AF9, ENL, ELL,* or *AF4* [120]. Work from our lab demonstrated that human HSPCs treated with low-dose, non-cytotoxic levels of etoposide and doxorubicin had increased DNA break frequency within the therapy-related breakpoint cluster region (BCR) of *MLL* [77]. Importantly, the concentrations of chemicals used in our study were modeled after the residual, low-dose concentrations found in the blood samples of cancer patients shortly following chemotherapy treatment [121,122]. This indicates that *MLL* is particularly sensitive to the effects of TOP2 poisons and, therefore, may explain why it is prevalent in many t-AML cases. Furthermore, we and others identified TOP2-mediated DNA breaks located at the therapy-related BCR of *MLL* [18,65,76,118,123,124,125], and mapped them to the region with a potential to form stable DNA secondary structure [76,77]. Recently, the TOP2 poison etoposide was shown to induce chromosome breakage and translocations involving *MLL, AF9, AF4, AF6*, and *ENL* in human HSPCs and lymphoblastoid cells [18]. Altogether, this emphasizes the sensitivity of this region and the role of TOP2 and its poisons on facilitating the mutagenic process that leads to t-AML in breast cancer and NHL patients.

### 5.2. Pediatric MLL-Rearranged AML

Multiple studies implicated maternal diet and occupational exposure in incidences of pediatric/infant AML. Specifically, studies focused on the role of both synthetic and natural TOP2 inhibitors and poisons in pediatric AML. Maternal exposure to TOP2 poisons such as bioflavonoids was focused on because roughly 80% of pediatric AML cases have *MLL* translocations, and those translocation events have breakpoints at locations that are highly similar to t-AML cases [126,127,128]. Maternal diet and occupational exposure are important factors to consider when determining causes of pediatric AML, as chemicals can cross the placenta and consequently affect TOP2 in the cells responsible for leukemia [129,130]. Further evidence supporting the formation of the *MLL* translocation in utero includes (1) identification of the translocation in neonatal blood spots of children diagnosed with AML, and (2) demonstration of monozygotic twins possessing identical AML translocations [131,132,133].

There are multiple chemicals and food items that are demonstrated/proposed to be TOP2 poisons. In terms of occupational exposures, benzene is of particular interest because it is a known carcinogen, with evidence supporting its role in leukemogenesis [134]. Additionally, benzene metabolites have anti-TOP2 capabilities [9,10,11], and data from our lab indicated that treatment of human HSPCs with low doses of benzene resulted in an increase of DNA breaks in the BCR of *MLL* [77]. An epidemiological study found that children whose mothers were exposed to benzene had about a two-fold increase in risk for AML [135]. Strong indications of the impact of maternal diet on the development of pediatric AML came from a 10-year study that found that children whose mothers had medium to high intake levels of TOP2 poison-containing food had a 10-fold higher risk for developing AML [128]. There is also strong evidence supporting the role of bioflavonoids in dietary-based TOP2 inhibition [12,136,137,138] and, therefore, leading to *MLL* translocations, despite conflicting opinions regarding the role of alcohol, tea, coffee, and cocoa in this process [139,140,141,142]. Dietary sources of bioflavonoids include soy-based products (genistein), fruits and vegetables (quercetin), and dietary supplements. Importantly, bioflavonoids can induce DNA cleavage at the same location in *MLL* and a partner gene as classic TOP2 poisons, and they can inhibit in vitro TOP2 activity to a similar extent as etoposide and doxorubicin [12]. Recent work demonstrated that quercetin and genistein can promote the formation of *MLL*/*AF9* translocations in hematopoietic stem cells [143]. Overall, there is strong support for the role of TOP2 inhibition, from occupational exposure and/or maternal diet, in promoting DNA cleavage of oncogenic loci, as well as the subsequent translocations that drive adult and pediatric AML.

### 5.3. RET-Driven Papillary Thyroid Cancer

In addition to the role of TOP2 in hematological malignancies, there is evidence to suggest TOP2 also facilitates the formation of oncogenic translocations in solid tumors. Papillary thyroid cancer (PTC) incidences increased dramatically since the 1970s according to the National Cancer Institute’s Surveillance, Epidemiology, and End Results (SEER) database [144,145,146]. While radiation exposure is linked with thyroid cancer, it does not appear to be the main cause with this rise in incidences [147,148]. Rather, increased exposure to environmental chemicals and chemotherapeutic agents is proposed to be the driving force in these cases.

Sporadic rearrangement-positive PTC cases are believed to be predominantly chemical exposure-related as 50–71% of these cases possess the *RET*/*PTC1* rearrangement [149,150,151]. On the other hand, in rearrangement-positive radiation-associated cases, the *RET/PTC3* rearrangement is more commonly the driver mutation [149,152,153]. Genes involved in the *RET/PTC1* and *RET/PTC3* rearrangements (*RET*, *CCDC6*, and *NCOA4*) are within known fragile sites [154], indicating that they are more prone to DNA breaks than other, non-fragile regions of the genome. This fragility and its direct role in the *RET/PTC1* translocation process was first demonstrated by our laboratory when non-malignant thyroid (HTori-3) cells were treated with low doses of fragile site-inducing chemicals and only the *RET/PTC1* rearrangement was observed [155].

Due to these early indications of chemical exposure-associated PTC cases, we further examined the contribution of chemicals such as benzene and/or chemotherapeutic agents, like TOP1 and TOP2 inhibitors/poisons, to incidences of PTC. Benzene is a carcinogen with anti-TOP2 properties and is often found in cigarette smoke, gasoline, and industrial emissions [9,10,11,156]. Low-dose exposure from these sources is sufficient for fragile site induction [156,157,158]. Interestingly, significantly more cases of PTC were observed in the area near the base of Mount Etna, possibly due to benzene exposure from volcanic eruptions [159,160]. PTC cases were also observed as secondary, treatment-related malignancies. Most notably, PTC was observed as a secondary cancer in patients treated with fragile site-inducing chemotherapeutic agents for cancers such as Hodgkin’s lymphoma, osteosarcoma, pediatric rhabdomyosarcoma, and others [161,162,163,164,165,166,167,168,169,170,171,172]. Based on these studies, there appears to be increasing evidence that chemical exposure contributes to incidences of PTC.

Recently, we observed a significant increase in the frequency of DNA breaks within the *RET* BCR in intron 11 when HTori-3 cells were treated with low-dose, non-cytotoxic levels of benzene, etoposide, and doxorubicin [173]. This suggests TOP2 plays a role in the translocation process of PTC. Furthermore, we showed that HTori-3 cells treated with fragile site-inducing chemicals or TOP1/TOP2 poisons generate DNA breaks within *RET* intron 11 that are predominantly distributed at or around predicted TOP1 and TOP2 cleavage sites [34]. *RET* intron 11 is capable of forming stable, DNA secondary structures [34,73]. Interestingly, chemical-induced breaks within this region were mapped to predicted TOP1 and TOP2 cleavage sites on these secondary structures. As topoisomerase enzymatic activity is enhanced by DNA secondary structures, this suggests that the fragility associated with *RET* intron 11 is due to the activity of topoisomerases at the DNA secondary structures [13,69,70,71]. Future research remains to be done to understand the role of TOP2 in facilitating oncogenic rearrangements in both hematological and solid cancers.

### 5.4. Other Diseases

TOP2 and proteins associated with processing TOP2 adducts were also reported to be involved in B-cell immunodeficiency [174], autism [54,63], autoimmune-related disorders [175], and neurodegeneration [176,177,178,179]. Properly functioning TOP2A is required for embryonic development in zebrafish and mice [180,181,182]. Even though organisms with TOP2B germline mutants can survive embryonic development, they die shortly after birth due to lack of proper neuronal differentiation, organization, and connections [183,184,185,186].

Heterozygous *TOP2B* mutants were recently identified in patients with B-cell immunodeficiency, and, using a murine model, Broderick et al. demonstrated increased DNA damage in mutant cells [174]. Many B-cell-specific transcription factor genes are relatively long and require TOP2B for efficient transcription, suggesting a pathogenesis mechanism for the *TOP2B* mutations and the role of TOP2B in B-cell development.

Recent studies found that topoisomerases help facilitate effective transcription of the genes linked with autism spectrum disorders (ASD). Interestingly, the ASD-associated genes were found to be particularly long and AT-rich [54,63], which begins to explain why topoisomerase activity is so critical for their transcription and subsequent expression. Additionally, multiple studies demonstrated that individuals with ASD have increased oxidative stress in their brains [187,188,189,190]. Since modified bases or mismatches can increase stability of TOP2ccs, the increase of oxidative stress-induced adducts may result in more TOP2-induced DSBs.

TOP2 is also associated with autoimmune disorders like insulin-dependent diabetes mellitus (IDDM). IDDM is classified as an autoimmune disorder because the beta cells that produce insulin are destroyed by T cells due to the presentation of autoantigens. Almost half of IDDM patients had TOP2A autoantibodies [175]. Macrophages release nitric oxide which causes extensive DNA damage in the islet cells, and leads to destruction of the nucleus and eventual presentation of nuclear proteins, like TOP2, as autoantigens [191]. Nitric oxide was recently shown to be a TOP2 poison and induced DNA breaks in a mouse model [192], indicating that the macrophage-released nitric oxide could generate TOP2-induced DNA damage in islet cells.

Furthermore, TOP2-associated diseases arise from mutations in the proteins that process TOP2 adducts and TOP2-induced DNA breaks, as demonstrated by the impact of these mutant proteins in neurons. TDP2 mutations cause neurological effects, specifically, seizures, ataxia, and intellectual disabilities. TDP2 mutations are also associated with defects in transcription and ineffective repair of TOP2-induced DNA breaks, which leads to incorrect transcript levels for proteins that are required for proper neurological function and neuronal organization of the cerebellum [176]. Mutations in MRE11 and ATM are also associated with neurodegenerative diseases. Both proteins are involved in the initial end processing of TOP2 adducts, and ATM also prevents excessive degradation during repair [4,193]. Individuals with mutations in MRE11 or ATM are diagnosed with ataxia telangiectasia-like disorders or classic ataxia telangiectasia, respectively. Common neurological symptoms of these disorders include ataxia, cerebellar degeneration, and seizures due to neuronal degeneration [177,178,179,194]. Similar to TDP2 mutations, MRE11 and ATM mutations could lead to increased DNA damage and improper protein levels in the neurons, leading to eventual neurodegeneration. Again, a fine balance exists between the promotion of efficient transcription in neurons by TOP2 and the detrimental effects of TOP2 adducts.

## 6. Concluding Remarks

We discussed how TOP2-induced DSBs are required for relieving torsional stress and resolving DNA structures during replication and transcription, as well as at TAD boundaries. Moreover, TOP2-induced breaks were shown to promote signal-induced transcription and facilitate dynamic chromatin contacts such as those between promoters and enhancers. In promoting these various processes, TOP2-associated DNA breaks could then be left unrepaired or repaired incorrectly, resulting in a variety of genomic mutations. TOP2 and the proteins associated with TOP2 end processing can directly and indirectly contribute to DNA fragility. Therefore, understanding the repercussions of impairing these proteins with environmental chemicals, dietary topoisomerase inhibitors, and/or chemotherapy regimens will allow us to mitigate exposure-related incidences of cancer in a timely manner.
